# TIM-family Proteins Promote Infection of Multiple Enveloped Viruses through Virion-associated Phosphatidylserine

**DOI:** 10.1371/journal.ppat.1003232

**Published:** 2013-03-28

**Authors:** Stephanie Jemielity, Jinyize J. Wang, Ying Kai Chan, Asim A. Ahmed, Wenhui Li, Sheena Monahan, Xia Bu, Michael Farzan, Gordon J. Freeman, Dale T. Umetsu, Rosemarie H. DeKruyff, Hyeryun Choe

**Affiliations:** 1 Division of Respiratory Diseases Children's Hospital Boston, Harvard Medical School, Boston, Massachusetts, United States of America; 2 New England Primate Center, Department of Microbiology and Immunobiology, Harvard Medical School, Boston, Massachusetts, United States of America; 3 National Institute of Biological Sciences, Beijing, China; 4 Division of Immunology, Children's Hospital Boston, Harvard Medical School, Boston, Massachusetts, United States of America; 5 Department of Medical Oncology, Dana-Farber Cancer Institute, Boston, Massachusetts, United States of America; National Institutes of Health, United States of America

## Abstract

Human T-cell Immunoglobulin and Mucin-domain containing proteins (TIM1, 3, and 4) specifically bind phosphatidylserine (PS). TIM1 has been proposed to serve as a cellular receptor for hepatitis A virus and Ebola virus and as an entry factor for dengue virus. Here we show that TIM1 promotes infection of retroviruses and virus-like particles (VLPs) pseudotyped with a range of viral entry proteins, in particular those from the filovirus, flavivirus, New World arenavirus and alphavirus families. TIM1 also robustly enhanced the infection of replication-competent viruses from the same families, including dengue, Tacaribe, Sindbis and Ross River viruses. All interactions between TIM1 and pseudoviruses or VLPs were PS-mediated, as demonstrated with liposome blocking and TIM1 mutagenesis experiments. In addition, other PS-binding proteins, such as Axl and TIM4, promoted infection similarly to TIM1. Finally, the blocking of PS receptors on macrophages inhibited the entry of Ebola VLPs, suggesting that PS receptors can contribute to infection in physiologically relevant cells. Notably, infection mediated by the entry proteins of Lassa fever virus, influenza A virus and SARS coronavirus was largely unaffected by TIM1 expression. Taken together our data show that TIM1 and related PS-binding proteins promote infection of diverse families of enveloped viruses, and may therefore be useful targets for broad-spectrum antiviral therapies.

## Author Summary

To infect cells, enveloped viruses typically utilize cellular receptors, which mediate specific, high-affinity interactions with the viral entry protein and prime the entry protein for subsequent steps in the viral entry process. Viral entry is also enhanced by attachment factors. Although less specific than receptors, attachment factors can alter the course of infection and thus severity of viral disease by increasing the infection efficiency of specific target cells. Here we observed that TIM proteins, a group of proteins that promote phagocytosis of apoptotic cells, can dramatically enhance the entry of a number of viruses, including Ebola, West Nile and dengue viruses, whereas they have little effect on the entry of other viruses. The inability of a virus to use TIM proteins may be due to the presence of an abundant, high-affinity receptor (Lassa fever virus), or because the TIM proteins direct virions to a non-productive internalization pathway (SARS coronavirus, influenza A virus). Mechanistically, TIM proteins appear to interact with enveloped viruses and apoptotic cells similarly by binding phosphatidylserine residues exposed on the viral and cellular membranes. Collectively our studies show that TIM proteins are attachment factors that can substantially improve the infection efficiency of a number of pathogenic viruses.

## Introduction

The entry of enveloped viruses is a multi-stage process. Following attachment, some viruses fuse to cells at the plasma membrane, whereas others are internalized through various endocytic routes and, primed by low pH or compartment-resident factors, fuse at the endo/lysosomal membranes. Viruses attach to the cell surface through the binding of their entry glycoproteins (GPs) to specific receptors/coreceptors and also through less specific interactions with various attachment factors [1]. While the distinction between attachment factors and *bona fide* receptors has not always been carefully made, receptors typically involve necessary, specific, and high-affinity interactions that can prime the viral entry protein for subsequent fusion steps. Attachment factors, in contrast, are typically interchangeable, involve less specific interactions, and serve primarily to localize the virus to its receptor(s) and other cofactors necessary for fusion.

A recent study reported that human TIM1 (hTIM1), a protein previously implicated as a receptor for the non-enveloped hepatitis A virus [2]–[4], also functioned as a receptor for the enveloped viruses Ebola (EBOV) and Marburg (MARV) [5]. This observation added hTIM1 to the long list of filovirus entry factors, which include β1-integrins [6], [7], the folic acid receptor alpha, which was later disputed [8]–[10], the TAM receptors Axl, Mer and Tyro [11], various C-type lectins [12]–[14] and the intracellular receptor Niemann-Pick C1 (NPC1) [15]–[17]. hTIM1 was identified by correlating gene expression patterns of 60 cancer cell lines with their permissiveness to EBOV entry [5].

The TIM protein family is composed of three members in humans (hTIM1, 3, and 4) and eight in mice (mTIM1-8) that are implicated in the regulation of innate and adaptive immune responses [18]. Based on expression, functional and structural data hTIM1, 3, and 4 are considered direct orthologs of mTIM1, 3, and 4, respectively [19], [20]. The ectodomain of TIM proteins includes an N-terminal variable immunoglobulin-like (IgV) domain and a stalk-like mucin domain that varies in length and O-glycosylation [18]. Importantly, the IgV domains of all hTIM proteins contain a high-affinity binding site for PS, a phospholipid constituent of eukaryotic membranes [21], [22]. Generally present on the cytosolic side of the plasma membrane lipid bilayer, PS flips to the outer leaflet upon the onset of apoptosis, where it acts as a so-called “eat-me” signal for professional phagocytes (macrophages and dendritic cells) as well as non-professional phagocytes (e.g., epithelial cells) [23]–[25]. Consistent with a PS receptor function, one important role of TIM proteins is to initiate PS-mediated engulfment of apoptotic cells and debris [21], [22], [26]. Although this role may be more prominent for TIM3 and TIM4, which are expressed on dendritic cell and macrophage sub-populations [21], [22], [26], [27], TIM1 is known to be expressed on various epithelial cells [5], [28], which can also assume phagocytic roles. TIM1 and TIM3 are further expressed on subsets of activated T-cells, where they act as costimulatory and coinhibitory molecules, respectively [29]–[31].

Several recent findings suggest that negatively-charged phospholipids like PS might play a role in mediating virus entry. PS was shown to be exposed on the membranes of various enveloped viruses, including Pichinde virus, vesicular stomatitis virus (VSV) and the intracellular mature virion form of Vaccinia virus [32], [33]. This is likely a common feature of most enveloped viruses, as virus-infected cells were shown to overexpress PS on their plasma membranes [32]–[34]. In addition, the entry of lentiviral pseudoviruses bearing the GPs of Sindbis (SINV), Ross River (RRV) and Baculo virus was enhanced in a PS-dependent manner by the TAM receptor tyrosine kinase Axl [35]. Axl has also been shown to promote EBOV and MARV entry [11]. Finally, an antibody targeting anionic phospholipids effectively rescued rodents from lethal challenges by either Pichinde virus or mouse cytomegalovirus, demonstrating *in vivo* contribution of anionic phospholipids to the infectivity of these viruses [33].

Because hTIM1 binds PS with high affinity [21] and various viruses contain PS on their virion surfaces [32], [33], we explored the possibility that the reported hTIM1-mediated EBOV entry [5] is PS dependent. Furthermore, using pseudoviruses, VLPs, and infectious viruses bearing the entry proteins of 19 viruses from 7 different families, we investigated the generality of PS-receptor usage and its underlying mechanisms. We observed that hTIM1 promoted infection by a range of viruses, including members of the filovirus, flavivirus, alphavirus and New World arenavirus families, and that this enhancement required the PS-binding activity of hTIM1. We further demonstrated that additional PS receptors, such as hTIM4 and hAxl enhanced the entry of most hTIM1-using pseudoviruses, and that the efficiency of this enhancement largely correlated with that observed with hTIM1. Finally, we explore and discuss the molecular mechanisms that contribute to the differential efficiency of viral PS receptor usage. Collectively our data suggest that hTIM1 and related proteins function as attachment factors for a full range of enveloped viruses.

## Materials and Methods

### Cells lines and plasmids

HEK293T (human embryonic kidney), Huh7 (human hepatocarcinoma), 3T3 (murine embryonic fibroblast) and MDCK (canine kidney) cells were grown at 37°C in DMEM supplemented with 10% fetal bovine serum (FBS), 1 mM sodium pyruvate and 1% penicillin/streptomycin (P/S). NPC1^−/−^ and NPC1^−/−^mNPC1 CHO cells (also referred to as M12 and wt8, respectively [36]) were kept at 37°C in DMEM/F12 with 5% FBS and 1% P/S. These cells were gifts from Daniel S. Ory at Washington University School of Medicine. C6/36 *Aedes albopictus* mosquito cells were maintained at 28°C in DMEM containing 10% FBS, 1 mM sodium pyruvate and 1% P/S.

Coding sequences of hTIM1, hTIM3 and hTIM4 (without signal peptides) were PCR amplified from human T-cell or macrophage cDNA libraries and cloned into the retroviral expression vector pQCXIX (BD Biosciences) downstream of the signal peptide of mouse angiotensin-converting enzyme 2 (ACE2) and a myc-tag. Their sequences were deposited to the Genbank under accession numbers JX049978, JX049979 and JX049980, respectively. hAxl cDNA (clone 5205825, Invitrogen) was purchased and similarly cloned into pQCXIX with an N-terminal myc-tag. The PS-binding deficient variant of hTIM1 (AA-hTIM1) was created by site-directed mutagenesis. It carries W112A and F113A mutations based on the previously-described equivalent mutations in other TIM proteins [19], [21]. The stalk-truncated Δ131-221 hTIM1 and Δ197-287 hTIM1 variants were obtained using deletion mutagenesis.

The plasmids encoding N-terminally myc-tagged human ACE2 (hACE2) and civet cat ACE2 were previously described [37]. Also previously described were the expression plasmids encoding the entry glycoprotein precursors of Zaire EBOV (Mayinga) and Lake Victoria MARV (Musoke), both lacking the mucin domains, Amapari virus (AMAV, BeAn 7063), Tacaribe virus (TCRV), Junín virus (JUNV, MC2), Machupo virus (MACV, Carvallo), Lassa fever virus (LASV, Josiah), lymphocytic choriomeningitis virus (LCMV, Armstrong), Chikungunya virus (CHKV, 37997), Eastern Equine Encephalitis virus (EEEV, FL91-4697), influenza A virus (FLUAV, H7: Rostock, N1: Puerto Rico), VSV (Indiana), SARS coronavirus (SARS-CoV; Tor2, GD and SZ) and West Nile virus (WNV, lineage 1, NY99) [37]–[43]. The precursor of the Oliveros virus (OLIV) entry protein was synthesized by GenScript based on Genbank ID AAC54654.1 and was cloned into pCAGGS.

### Assessment of receptor expression

The expression of various receptors was assessed with monoclonal antibodies and detected using the Accuri C6 flow cytometer (BD Biosciences). To measure endogenous hTIM1 expression, cells were stained with mouse anti-hTIM1 antibody (clone 219211, R&D Systems) or purified mouse Fc (mFc) as a negative control. Primary antibody binding was detected with a phycoerythrin (PE)-conjugated goat anti-mouse antibody (Jackson ImmunoResearch Laboratories). To assess endogenous mouse TIM1 (mTIM1) expression, cells were stained with anti-mTIM1-PE, a PE-conjugated rat anti-mTIM1 antibody (clone RMT1-4, BioLegend) or with anti-mIFNγ-PE, a PE-conjugated rat anti-mouse interferon gamma antibody (BioLegend) used as negative control. Cells transfected or transduced to express exogenous receptors were stained with various antibodies. While the antibody used to detect wildtype (wt) hTIM1 and the AA-hTIM1 variant was the anti-hTIM1 antibody described above, that used in the hTIM1 stalk truncation experiment was anti-hTIM1 antibody 3D1, which specifically recognizes the IgV head domain of hTIM1 [21]. Expression of N-terminally myc-tagged hAxl, hTIM3/4, hACE2 and civet cat ACE2 was assessed using anti-myc antibody 9E10.

### MLV pseudovirus and WNV VLP entry assays

Pseudoviruses bearing various viral entry proteins were produced in 293T cells as described [39] by calcium-phosphate transfection of a retroviral vector pQCXIX (BD Biosciences) encoding eGFP together with two other plasmids, separately encoding a viral entry protein and the Moloney murine leukemia virus (MLV) gag and pol. For FLUAV H7N1, an additional plasmid encoding N1 neuraminidase (Puerto Rico) was co-transfected [41]. Pseudovirus-containing culture supernatants were harvested at 32–34 h post-transfection, filtered through a 0.45 µm PES membrane, stored at 4°C, and used within 2 weeks. WNV VLPs were produced and harvested in the same way after co-transfecting a plasmid encoding the WNV structural proteins (capsid and entry glycoproteins prM and E) and a WNV replicon encoding the non-structural proteins NS1-5 and GFP [43].

For infection, cells were plated on poly-lysine-coated (293T) or uncoated 48-well plates and incubated at 37°C with pseudovirus- or VLP-containing supernatants diluted to yield comparable levels of infection. In order for virus entry enhancement not to be limited by virus titers, cells were generally incubated with virus supernatants for less than 1 hour, unless mentioned otherwise. Supernatants were then replaced with fresh medium, incubated for one (293T) or two (3T3, Huh7 and NPC1^−/−^) days to allow for eGFP reporter expression and infection levels were assessed by measuring GFP fluorescence with the Accuri C6 flow cytometer (BD Biosciences). Independent experiments were performed with independent pseudovirus and VLP preparations. In addition, all pseudoviruses and VLPs used in a given experiment were produced in parallel.

### Infection assays with replication-competent viruses

Lyophilized infectious TCRV (TRVL 11573), passaged in suckling mice and Vero cells, as well as RRV (T-48) and SINV (Ar-339), both passaged in suckling mice, were purchased from ATCC, and resuspended in PBS according to the instruction. Type 2 infectious dengue virus (DENV, New Guinea C) was obtained by passaging in C6/36 *Aedes albopictus* mosquito cells. Virus-containing culture supernatants were 0.45 µm-filtered, stored at −80°C and virus titers determined using plaque assays in baby hamster kidney cells as described [44]. Infectious FLUAV (H1N1, A/PR/8/34) was propagated in MDCK cells [45], 0.45 µm-filtered and virus titers assessed as tissue culture infectious dose 50 (TCID50) in cultured human macrophages using CellTiter-Glo One solution (Promega). For infection, cells were incubated for 1–6 h at 37°C with viruses serially diluted in DMEM containing 10% FBS, washed and supplemented with fresh medium. At indicated days post-infection, cells were detached by trypsinization, washed, fixed with 1% formaldehyde in PBS and permeabilized with 0.1% saponin in PBS containing 2% goat serum. Cells were then stained with immune ascitic fluids (ATCC) for TCRV, RRV and SINV, anti-FLUAV antibody C111 (Clontech), or anti-DENV antibody 2H2 (Millipore) [46] followed by an Alexa 649- or PE-conjugated goat anti-mIgG antibody (Jackson ImmunoResearch Laboratories) and analyzed by flow cytometry.

### Internalization assays

VP40-GFP VLPs were produced in 293T cells by co-transfecting a pCAGGS-based plasmid encoding a previously described GFP-fusion version of the EBOV VP40 matrix protein [47], a gift from Christopher F. Basler at Mount Sinai School of Medicine, with a plasmid encoding mucin-domain-deleted EBOV GP at 3∶1 ratio. To make VP40-GFP VLPs bearing no entry protein, the VP40-GFP plasmid was similarly cotransfected with an empty plasmid. VLP-containing culture supernatants were harvested at 36 h post-transfection and cell debris removed by two consecutive centrifugations at 900 g. The presence of fluorescent filovirus-like particles in the supernatants was confirmed by fluorescence microscopy.

MLVgag-GFP pseudovirions were produced similarly as described for the non-fluorescent pseudoviruses, except that 25% of the MLV *gag-pol* plasmid DNA was replaced with plasmid DNA encoding a MLV gag-GFP fusion protein [48], a gift from Walther Mothes at Yale University School of Medicine. Also, pQCXIX-eGFP was replaced with the same vector encoding a non-fluorescent protein. Virus supernatants were harvested at 34–36 h post-transfection, 0.45 µm-filtered, purified by ultracentrifugation (SW40Ti rotor, 70'000 g for 2 h at 10°C) and resuspended in a small volume of DMEM containing 10% FBS.

To assess VLP/pseudovirion internalizaton, cells were incubated for 2–6 h at 37°C with VP40-GFP VLPs, with MLVgag-GFP virions normalized for MLV reverse-transcriptase (RT) activity or with mock supernatants obtained from cells transfected with a plasmid expressing eGFP alone. Uninternalized VLPs and virions were removed with two 1 min acid-washes (200 mM glycine, 150 mM NaCl, pH 3.0) followed by trypsinization at 37°C for 15 min. Internalization of VLPs or pseudovirions was assessed by flow cytometry.

### Entry assays with VP40-β-lactamase VLPs and macrophages

VLPs made with EBOV VP40 matrix proteins fused to β-lactamase (Bla) and bearing EBOV GP, LASV GP or no GP were produced as described above for VP40-GFP VLPs. The VP40-Bla fusion construct was a gift from Paul Bates at University of Pennsylvania [8]. Naive peritoneal macrophages were obtained from wildtype BALB/cBYJ mice. Briefly, peritoneal cavity cells were plated in 12-well plates at 10^6^ per well and incubated for 1 h at room temperature to let macrophages adhere. After removing non-adherent cells by washing twice in PBS/2% FBS, macrophages were incubated overnight at 37°C in RPMI containing 10% FBS, 1% P/S and 50 µM β-mercaptoethanol and infected the following day for 2 h at 37°C with VP40-Bla VLPs. Infected cells were detached by scraping in trypsin/EDTA, washed and loaded with the Bla substrate CCF2-AM, as previously described [49]. The conversion of substrate by cytoplasmic esterases and Bla, which reflects VP40-Bla VLP entry, was detected using the LSR II flow cytometer (BD Biosciences).

### Liposome blocking assays

1,2-diacyl-sn-glycero-3-phospho-L-serine (PS), 1,2-diacyl-sn-glycero-3-phosphocholine (PC) and 1,2-diacyl-sn-glycero-3-phosphoethanolamine (PE) were purchased from Sigma and resuspended at 10 mg/ml in chloroform. Aliquots of PC alone or PC mixed with PS or PE at 1∶1 molar ratios were dried under argon and then in a SpeedVac for 1 h, followed by hydration in PBS at a concentration of 1 mM total lipids. Liposomes were made by sonicating these milky lipid suspensions to clarity, stored at 4°C, and used within a week. For both entry and internalization blocking assays, liposome preparations were diluted in the appropriate medium containing 10% FBS and preincubated with cells for 20–30 min at room temperature. Pseudoviruses or VLPs were then added and culture plates shifted to 37°C for infection. Independent experiments were performed with independent liposome preparations.

### Accession numbers

Relevant SwissProt accession numbers are Q96D42 (hTIM1), Q8TDQ0 (hTIM3), Q96H15 (hTIM4), P30530 (hAxl), Q9BYF1 (hACE2), P02786 (hTfR1) and O15118 (NPC1).

## Results

### hTIM1 promotes pseudovirus and VLP entry mediated by a range of viral entry proteins

**Figure 1:**
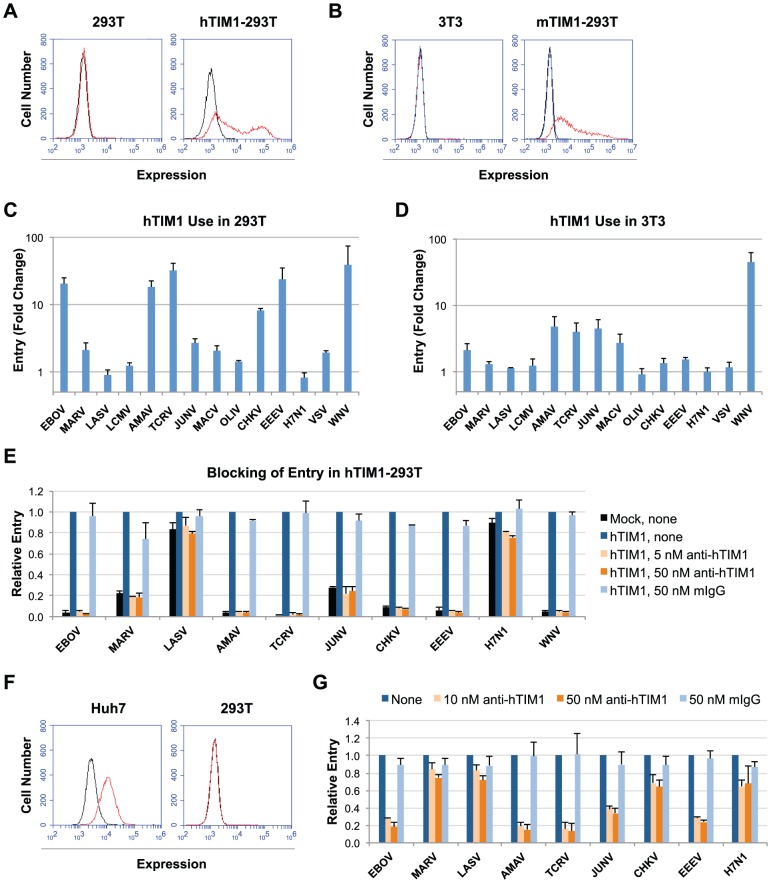
hTIM1 promotes infection mediated by a range of viral entry proteins. (A) Human 293T cells do not express TIM1. 293T and control cells were stained with anti-hTIM1 antibody (red) or with mFc (black). (B) Murine 3T3 cells do not express TIM1. 3T3 and control cells were stained with anti-mTIM1-PE antibody (red). Unstained cells (black) and cells stained with anti-mIFNγ-PE (blue) served as negative controls. (C) Exogenous hTIM1 increases the entry of various pseudoviruses in 293T cells. 293T cells were transfected with plasmids expressing hTIM1 or, as a negative control, hACE2. 48 h later cells were infected with MLV pseudoviruses or WNV VLPs, both carrying the GFP reporter gene. The following day, GFP expression was quantified by flow cytometry. Fold changes in entry were calculated by dividing mean fluorescence intensity observed in hTIM1-expressing 293T cells by those in hACE2-expressing 293T cells. Figure shows mean+SD from three independent, duplicated experiments. (D) hTIM1 usage by pseudoviruses was similarly assessed in 3T3 cells transduced with hTIM1 or hACE2. (E) Entry inhibition by an anti-hTIM1 antibody parallels hTIM1 use by pseudoviruses. 293T cells transduced with hACE2 (mock) or hTIM1 were preincubated for 30 min at room temperature with medium alone (none), the anti-hTIM1 antibody 3D1 or mIgG, and infected with pseudoviruses overnight in the presence of the respective blocking agents. Infection levels were normalized to those of untreated hTIM1-expressing cells. Figure shows mean+SD from two independent, duplicated experiments. (F) Human Huh7 cells express high levels of TIM1. Huh7 cells and control cells were stained for TIM1 expression as in A. (G) An anti-hTIM1 antibody inhibits entry of various pseudoviruses in Huh7 cells. Huh7 cells were incubated with antibodies and infected as in E. Infection levels were assessed 48 h later as in C and normalized to those of untreated cells. Figure shows mean+SD from three independent, duplicated experiments.

We first tested the specificity of viral use of hTIM1 in two cell lines that express little or no endogenous TIM1: human 293T and murine 3T3 cells (Figs. 1A, B). Cells engineered to overexpress hTIM1 or hACE2, a control receptor, were infected with a panel of 14 MLV pseudoviruses bearing the GPs of the filoviruses EBOV and MARV, the arenaviruses LASV, LCMV, AMAV, TCRV, JUNV, MACV and OLIV, the alphaviruses CHKV and EEEV, the orthomyxovirus FLUAV (H7N1), the rhabdovirus VSV or the coronavirus SARS-CoV. In addition, cells were infected with VLPs bearing the entry proteins of WNV, a member of the flavivirus family. As shown in Figures 1C and S1, relative to control cells, many pseudoviruses infected hTIM1-expressing 293T cells more efficiently. The entry of EBOV, AMAV, TCRV and EEEV pseudoviruses as well as WNV VLPs was strongly increased by hTIM1 (over 15 fold), that of CHKV considerably (8 fold) and that of MARV, JUNV, MACV and VSV moderately (2–5 fold). The entry of the remaining pseudoviruses tested - LASV, LCMV, OLIV, H7N1 and SARS-CoV - was increased by less than two-fold, with LASV, H7N1 and SARS-CoV being the least affected. Unlike in 293T cells, hTIM1 overexpression in 3T3 cells had a less dramatic impact on viral entry (Figs. 1D and S1): Only WNV VLPs showed a strong increase in entry in hTIM1-expressing 3T3 cells relative to control 3T3 cells, while EBOV, AMAV, TCRV, JUNV and MACV pseudoviruses showed a moderate increase. In both experiments virus titers were not limiting for non-hTIM1-using pseudoviruses, since longer infection times yielded much higher levels of entry in the control cells (Figs. S1B, D). Together these data suggest that hTIM1 supports the entry of a wide range of pseudoviruses, in particular those for which no high-affinity cell surface receptor has been identified. In addition, the effect of hTIM1 is dependent on the cellular background in which the experiment is performed.

To further assess the role of TIM1, we tested the ability of an anti-hTIM1 antibody to inhibit infection. As shown in Figure 1E in TIM1-expressing 293T cells the mouse monoclonal anti-hTIM1 antibody 3D1 [21] inhibited the entry of each pseudovirus to the same extent as its entry had been enhanced by hTIM1. Similar efficient inhibition was observed in Huh7 cells, a human cell line with high endogenous hTIM1 expression (Figs. 1F,G): Anti-hTIM1 antibody 3D1 inhibited the entry of EBOV, AMAV, TCRV and EEEV pseudoviruses by over 70% at a 10 nM concentration, indicating that hTIM1 serves as a major entry factor for these viruses in Huh7 cells. JUNV pseudovirus entry was also considerably inhibited (by 60%), suggesting that hTIM1 can contribute to the entry of this virus in Huh7 cells, although JUNV predominantly uses human transferrin receptor 1 (hTfR1) in other cells [42], [50], [51]. In contrast, the other pseudoviruses tested were only moderately affected by the presence of anti-hTIM1 3D1. While results of these entry blocking experiments are overall consistent with the gain-of-entry experiments in 293T cells (Fig. 1C), there are a few exceptions. For instance, MARV and CHKV pseudovirus entry was blocked less efficiently than expected in Huh7 cells (Fig. 1G) and Huh7 cells were not efficiently infected by WNV VLPs (data not shown).

### hTIM1 enhances infection of various replication-competent viruses

**Figure 2:**
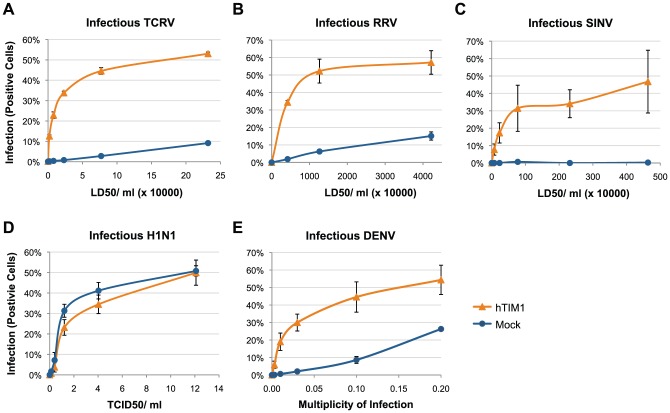
hTIM1 increases the infection of diverse replication-competent viruses. 293T cells transduced with hTIM1 or hACE2 (mock) were infected for 1–6 h with infectious TCRV (A), RRV (B), SINV (C), H1N1 (D), or DENV (E) at increasing titers. Cells were trypsinized the following day (D and E) or 2 days later (A, B and C), stained either with immune ascitic fluids (A, B and C) or with anti-FLUAV (D) or anti-DENV (E) antibodies. Figures show a representative of the two duplicated experiments (A) or mean ± SD of two independent, duplicated experiments (B–E). LD50: Lethal dose 50, as determined in suckling mice at ATCC.

We then confirmed the role of hTIM1 in virus infection, using replication-competent viruses. To circumvent the need for BSL3 or 4 laboratories, required for infectious MACV, JUNV, CHKV, EEEV or WNV, we chose TCRV to represent the arenavirus family, RRV and SINV to represent the alphavirus family and DENV to represent the flavivirus family. In addition, FLUAV H1N1 was used as a negative control. When hTIM1-293T cells and the control hACE2-293T cells were infected with serially diluted viruses, infection levels were markedly enhanced in hTIM1-293T cells across the board, except for H1N1 (Fig. 2), which is consistent with the pseudovirus entry data shown in Figure 1. Of note, the magnitude of infection enhancement was generally greater at low virus titers, implying that TIM1 may play a more important role when virus titers are limiting, for example at the initial phases of infection. This initial advantage conferred by hTIM1 was further amplified during the first few cycles of replication (Fig. S2). As a surrogate for replication-competent EBOV we used EBOV VP40-based VLPs [8] bearing the wildtype EBOV entry protein, and confirmed that hTIM1 efficiently enhances their entry (data not shown).

### hTIM1-mediated enhancement of pseudovirus and VLP entry is PS dependent

**Figure 3:**
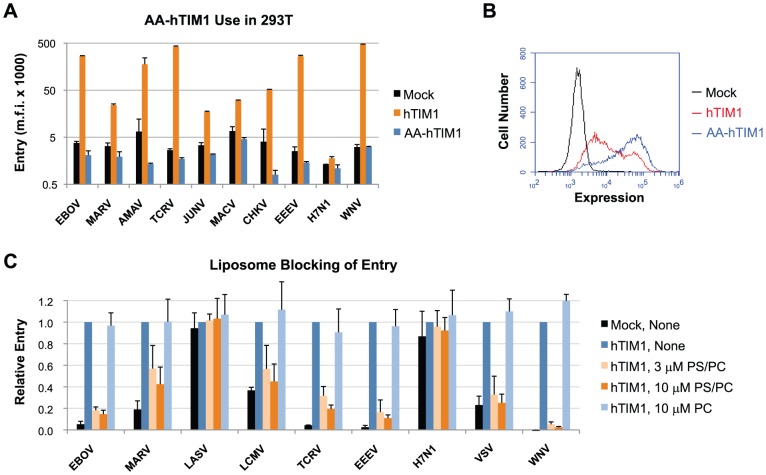
hTIM1-mediated entry of pseudoviruses and VLPs is PS dependent. (A) A hTIM1 variant deficient in PS binding (AA-hTIM1) does not support viral entry. 293T cells were transduced with hACE2 (mock), hTIM1 or AA-hTIM1, and infected 2 days later with the indicated pseudoviruses or WNV VLPs. Entry was determined by GFP-expression measured by flow cytometry. Figure depicts representative results from one of three independent, duplicated experiments. M.f.i.: mean fluorescent intensity. (B) In parallel with the infection in A, expression levels of hTIM1 and AA-hTIM1 were assessed as in Figure 1A. (C) hTIM1-mediated virus entry enhancement is efficiently blocked by PS-containing liposomes. 293T cells transduced with hACE2 (mock) or hTIM1 were preincubated for 20 min at room temperature with medium alone (none) or with liposomes consisting of either 50% PS and 50% PC (PS/PC) or PC alone (PC). Pseudoviruses or WNV VLPs were then added for a 30 min infection at 37°C, after which unbound liposomes and viruses were washed away and cells supplemented with fresh medium. Entry was quantified as in A and normalized to those of untreated hTIM1-expressing cells. Figure shows mean+SD of three independent, duplicated experiments.

hTIM1 is a receptor involved in the PS-mediated uptake of apoptotic cells [21]. To test the hypothesis that hTIM1 may broadly increase viral entry because it binds PS on viral membranes rather than to specific viral glycoproteins, we compared viral usage of a hTIM1 variant defective in binding PS with that of wt hTIM1. The mutant variant, hereafter referred to as AA-hTIM1, has two mutations in the PS-binding pocket that are known to nearly abrogate PS binding [19], [21]. Indeed, when these receptors were overexpressed in 293T cells we found that AA-hTIM1 was unable to enhance the entry of any pseudovirus tested (Fig. 3A) despite efficient AA-hTIM1 expression (Fig. 3B). This indicates that viral hTIM1 usage is PS-dependent.

To further assess the role of PS, we tested whether various liposomes are able to block hTIM1-mediated viral entry (Fig. 3C). Consistent with a PS-dependent mechanism, liposomes consisting of 50% PS and 50% PC, but not those consisting of PC alone, efficiently blocked the entry of all hTIM1-using pseudoviruses into hTIM1-expressing 293T cells at 3 µM concentration. In contrast, the entry of LASV and H7N1 pseudoviruses, whose entry is not enhanced by hTIM1, were not affected by either liposomes. Unexpectedly, PE-containing liposomes were also able to inhibit hTIM1-medated viral entry (Fig. S3). This observation raises the possibility that PE, another marker of apoptotic cells [52], which shares structural similarities with PS but is not negatively charged at physiological pH, may also play a role in viral hTIM1 usage.

### TIM1-mediated virion internalization is independent of viral entry proteins

**Figure 4:**
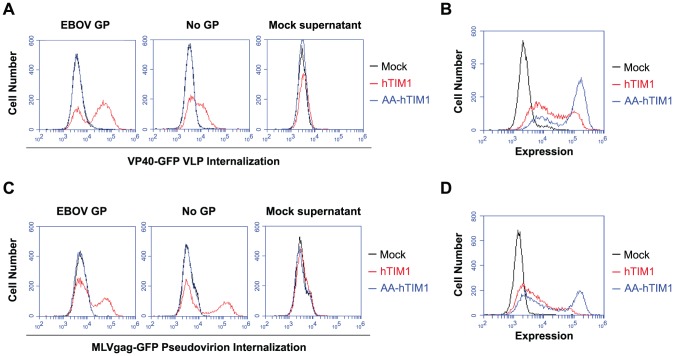
Viral entry proteins are not required for hTim1-mediated internalization into cells. (A) VP40-GFP VLPs lacking any viral entry protein are bound and internalized by hTIM1. 293T cells transduced with hACE2 (mock), hTIM1 or AA-hTIM1 were incubated for 6 h with VP40-GFP VLPs bearing EBOV GP or no GP. Being inherently fluorescent, these VLPs can be detected independently of whether they undergo fusion, in contrast to the pseudoviruses and VLPs used in previous figures. The supernatant of cells expressing only GFP (mock supernatant) served as negative control. Uninternalized VLPs were detached by acid-stripping and extensive trypsinization, after which internalized VLPs were detected by flow cytometry. Figure depicts results representative of three independent experiments performed in duplicates. (B) In parallel with the infection in A, expression levels of hTIM1 and AA-hTIM1 were assessed as in Figure 1A. (C) MLVgag-GFP pseudovirions lacking viral GPs are bound and internalized at least as efficiently as those bearing EBOV GP. 293T cells transduced with hACE2 (mock), hTIM1 or AA-hTIM1 were infected for 6 h with purified and RT-activity normalized MLVgag-GFP pseudovirions. The supernatant of cells expressing only GFP (mock supernatant) served as negative control. Cell surface-bound pseudovirions were detached by acid-stripping and extensive trypsinization, after which internalized virions were detected by flow cytometry. Data shown are representative of three independent experiments performed in duplicates. (D) In parallel with the infection in C, expression levels of hTIM1 and AA-hTIM1 were assessed as in Figure 1A.

PS dependency of viral hTIM1 usage implies that virions lacking any viral entry protein should also bind to and internalize into the intracellular compartments of hTIM1-expressing cells. To test this we took advantage of GFP-fused matrix proteins [47], [48] that allow, when incorporated into the virions, the detection of prefusion stages of viral entry. As shown in Figures 4A and B, when 293T cells expressing hTIM1, AA-hTIM1 or a control receptor were infected with EBOV VP40-matrix-based VLPs (VP40-GFP VLPs), those bearing no GPs were readily internalized by hTIM1, provided that the TIM1 PS-binding domain was functional. Although EBOV GP-bearing VP40-GFP VLPs appeared to be internalized more efficiently than those lacking GP, this bias is most likely due to the fact that the latter are released 3 to 5 times less efficiently from the producer cells [53], [54]. Consistent with this explanation, when the internalization experiment was repeated with RT-activity normalized MLVgag-GFP virions, we obtained even higher internalization efficiencies for GP-free than EBOV GP-bearing virions (Figs. 4C, D). Thus, results presented in Figure 4 demonstrate that virions bind to and internalize via hTIM1 in a manner that is independent of specific viral entry proteins, but instead is dependent on components of the viral membrane.

### Differing mechanisms underlie the inability of hTIM1 to promote infection of some pseudoviruses

**Figure 5:**
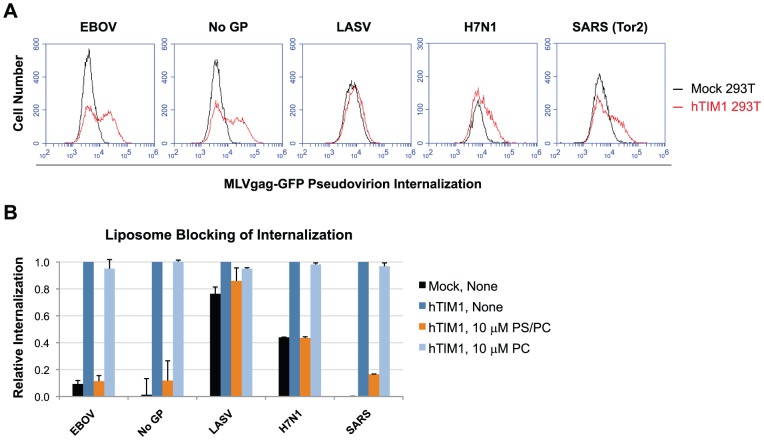
TIM1-mediated pseudovirus internalization does not always coincide with TIM1-mediated entry enhancement. (A) Some pseudoviruses that do not use hTIM1 for productive entry are still efficiently internalized by hTIM1. Mock- or hTIM1-transduced 293T cells were infected for 2 h with purified, RT-activity normalized MLVgag-GFP pseudovirions. Uninternalized virions were detached by acid-stripping and extensive trypsinization, after which internalized virions were detected by flow cytometry. Data shown are representative of two independent experiments performed in duplicates. (B) hTIM1-mediated pseudovirus internalization is blocked by PS-containing liposomes. Mock- or hTIM1-transduced 293T cells were preincubated for 20 min at room temperature with medium alone (none), or with liposomes consisting of either 50% PS and 50% PC (PS/PC) or PC alone (PC). MLVgag-GFP pseudovirions, prepared as in A, were then added for a 2 h infection at 37°C, after which bound virions were detached and internalized virions were detected as in A. Figure shows mean+SD of two independent, duplicated experiments.

**Figure 6:**
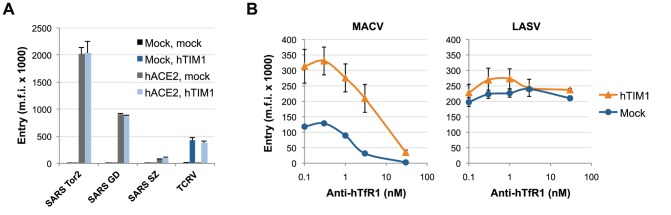
Receptor affinity does not determine the use of hTIM1 by SARS-CoV and MACV pseudoviruses. (A) 293T cells were transduced with empty vector (mock) or hACE2, the receptor for SARS-CoV, followed by a second transduction with empty vector or hTIM1. These cells expressing hACE2 and/or hTIM1 were then infected with pseudoviruses carrying the GFP-reporter gene and the entry glycoprotein of the indicated SARS-CoV isolates that use hACE2 with varying efficiency [37]. Comparable titers of these pseudoviruses were confirmed by infecting cells expressing civet cat ACE2, which is efficiently used by all SARS-CoV isolates [37]. Infection levels were assessed the following day by measuring GFP expression. Shown are representative results of one of three experiments performed in duplicates. (B) 293T cells transduced with hTIM1 or hACE2 (mock) were preincubated for 20 min at room temperature with increasing concentrations of anti-hTfR1 antibody ch128.1. MACV or LASV pseudoviruses were then added for a 1 h infection at 37°C, after which cells were washed and supplemented with fresh antibody-containing medium. Infection levels were assessed the following day as in A. Figure shows mean ± SD from two independent, duplicated experiments.

We next tested whether the observation that GP-free MLVgag-GFP virions are readily internalized by hTIM1 could be extended to the same virions bearing the GPs of LASV, H7N1 and SARS-CoV, which had been unaffected by hTIM1 expression in the entry assays that rely on a post-fusion readout (Figs. 1 and S1). As shown in Figure 5A, the internalization of H7N1 and SARS MLVgag-GFP virions, normalized for RT-activity, considerably increased in the presence of hTIM1, albeit less than that of EBOV and GP-free virions. We cannot, however, exclude the possibility that the SARS and H7N1 MLVgag-GFP virions that internalized via hTIM1, are those carrying fewer entry proteins on the their surface. Nonetheless, this internalization was blocked by PS-containing liposomes, but not by those consisting of only PC (Fig. 5B). These findings indicate that hTIM1 promotes the PS-dependent internalization of H7N1 and SARS-CoV virions without leading to productive infection (compare with Figs. 1C, 6A and S1E). In contrast, internalization of LASV MLVgag-GFP virions was only minimally increased by hTIM1 (Fig. 5A) and was not blocked by PS-containing liposomes (Fig. 5B), suggesting that the molecular mechanism responsible for the lack of hTIM1-mediated entry for LASV is distinct from those of H7N1 and SARS-CoV.

As suggested by Morizono et al. [35], PS receptor usage by viruses may be influenced by their affinity for other cell surface receptors. For instance, LASV internalization via alpha dystroglycan, its primary receptor, may be too efficient for PS receptors to compete with. We tested this receptor-affinity hypothesis using the GPs from various SARS-CoV isolates with differing affinities for the same receptor. Tor2, isolated from the major SARS-CoV outbreak, GD from a minor one, and SZ, from a reservoir species civet cat, respectively show high, moderate and low affinity to hACE2 [37]. As shown in Figure 6A, when 293T cells expressing hACE2, with or without hTIM1, were infected with pseudoviruses bearing these GPs, infection levels reached by these three pseudoviruses corresponded to their reported affinities to hACE2. However, no TIM1-mediated entry increase was observed with any of these SARS-CoV GPs, while the entry of the control pseudovirus (TCRV) was substantially enhanced. These results indicate that receptor affinity cannot explain the inability of hTIM1 to promote infection in the case of SARS-CoV. To further test the receptor-affinity hypothesis we assessed whether the blocking of hTfR1, a high-affinity receptor for MACV [42], might promote MACV's TIM1 usage. However, as shown in Figure 6B, the entry of MACV pseudovirus into TIM1-expressing 293T cells was as strongly inhibited by the anti-hTfR1 antibody ch128.1 [51] as in control cells. Thus, limiting the availability of the high affinity receptor does not increase TIM1 usage.

### Viral usage of the PS receptors hAxl and hTIM4 parallels that of hTIM1

**Figure 7:**
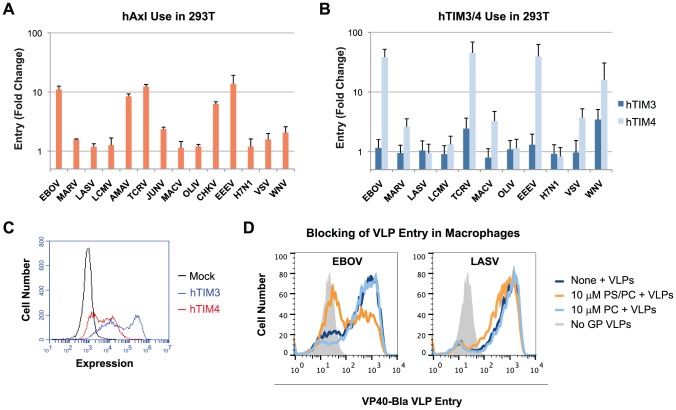
Other PS-binding receptors similarly promote entry of hTIM1-using pseudoviruses. (A) Exogenous hAxl usage in 293T cells. 293T cells were transfected with hAxl or hACE2, infected with pseudoviruses or WNV VLPs, both carrying a GFP reporter gene, and infection levels were assessed the following day by measuring GFP expression by flow cytometry. Fold changes in entry were calculated by dividing mean fluorescence intensity observed in hAxl-expressing 293T by those of hACE2-expressing 293T cells. Figure shows mean+SD from three independent, duplicated experiments. Note that the entry of WNV VLPs is only little increased by hAxl. (B) Exogenous hTIM3 and hTIM4 use in 293T cells. 293T cells were transfected with plasmids expressing hTIM3, hTIM4 or hACE2, infected with the indicated pseudoviruses or WNV VLPs and infection levels were assessed as in A. Shown are mean+SD from three independent experiments. Note that, unlike hTIM1 and hTIM4, hTIM3 only weakly enhanced infection of TCRV pseudoviruses and WNV VLPs. (C) Both hTIM3 and hTim4 are expressed efficiently. The same cells as in B were stained with anti-myc tag antibody. Figure shows representative results of one of three independent experiments. (D) PS receptors contribute to EBOV VLP entry in macrophages. Mouse peritoneal macrophages plated the previous day were preincubated for 30 min at room temperature with medium alone (none) or with liposomes consisting of 50% PS and 50% PC (PS/PC) or PC alone (PC). VP40-Bla VLPs bearing the entry proteins of EBOV, LASV, or no GP (negative control) were then added for a 2 h infection at 37°C, after which cells were detached, washed, loaded with the Bla substrate CCF2-AM, washed again and analyzed by flow cytometry. Figure is representative of two experiments performed in duplicates.

Since a number of human PS receptors have been described [25], we sought to determine if PS receptors other than hTIM1 have a similarly broad impact on viral entry. For example, hAxl was recently shown to enhance the infection mediated by the entry proteins of SINV, RRV and Baculovirus in a PS-dependent manner by binding the serum proteins Gas-6 and Protein S, which in turn bind PS displayed on these viruses' membranes [35]. As shown in Figure 7A, the infection of 293T cells expressing exogenous hAxl with the panel of pseudoviruses and VLPs used in Figure 1 yielded a pattern almost identical to that observed with hTIM1. One exception, however, was that WNV VLP entry was not much enhanced by hAxl, indicating that Gas-6 and Protein S from FBS might bind PS differently compared to TIM proteins. We then tested whether hTIM3 and 4, also shown to be PS receptors [21], [22], similarly enhance viral entry. Again, 293T cells expressing hTIM3, hTIM4 or a control receptor were infected with various pseudoviruses and WNV VLPs (Figs. 7B, C). While hTIM4 expression resulted in a pattern of entry enhancement similar to that of hTIM1, hTIM3 showed only moderate support of TCRV pseudovirus and WNV VLP entry. The mechanisms underlying inefficient viral TIM3 usage seem to be complex (see Fig. S4 and Supplementary Text S1). Collectively, these data indicate that different PS receptors tend to enhance infection of the same viruses - although not every virus uses every PS receptor - and suggest that PS receptors other than TIMs and Axl are also likely to increase the entry of a wide range of viruses using a common mechanism.

### Contribution of PS receptors to viral entry in physiological target cells

Experimental infections in animal models have shown that macrophages are early targets for the replication of several viruses [55], [56]. We therefore assessed the contribution of PS receptors to viral entry in mouse peritoneal macrophages, which are known to express TIM4 [21] and other PS receptors. To be able to detect viral entry instantaneously, we used VLPs consisting of EBOV VP40 matrix proteins fused to β-lactamase (VP40-Bla VLPs [8]) for infection. As shown in Figure 7D, the entry of VP40-Bla VLPs bearing EBOV GP was inhibited by 45% in the presence of PS-containing liposomes at 10 µM, reflecting as expected that other host cell molecules also play a role in virus infection. In contrast, the entry of the same VLPs bearing LASV GP was only little inhibited (by 8%). The blocking effect was specific for PS, as liposomes consisting of only PC did not inhibit either VLPs. These data are consistent with the notion that PS receptors can help potentiate viral infection in macrophages.

### hTIM1-mediated EBOV pseudovirus entry is dependent on NPC1

**Figure 8:**
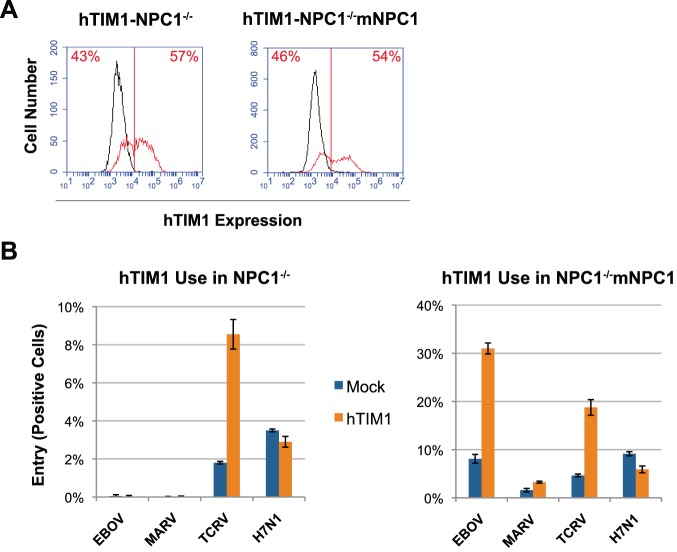
hTIM1-mediated EBOV and MARV entry is dependent on the presence of the intracellular receptor NPC1. CHO cells lacking NPC1 (NPC1^−/−^) and the same cells stably expressing mouse NPC1 (NPC^−/−^mNPC1) were mock transduced or transduced to express hTIM1. (A) Cells were assessed for hTIM1 expression levels using anti-hTIM1 antibody as in Figure 1A. (B) Cells were infected for 0.5–1 h with EBOV or MARV pseudoviruses, which were concentrated 3–5 fold by ultracentrifugation, or with unconcentrated TCRV or H7N1 pseudoviruses as positive and negative controls for TIM1 use. Infection levels for all viruses were assessed 2 days after infection by measuring GFP expression. Regardless of hTIM1 expression, NPC1^−/−^ cells showed no GFP positive cells with either EBOV or MARV infection when inspected by fluorescence microscopy. Figure depicts representative results of one of three experiments performed in duplicates.

EBOV and MARV entry was recently shown to critically depend on NPC1, a cholesterol-transporting protein located in the endo/lysosomal compartment [15]–[17]. To test whether hTIM1 usage by EBOV and MARV is dependent on NPC1, we used NPC1-null CHO cells (NPC1^−/−^), as well as NPC1-null CHO cells engineered to overexpress mouse NPC1 (NPC1^−/−^mNPC1), which supports EBVO entry [36]. As expected based on our earlier results (Fig. 1), EBOV pseudovirus entry, and to a lesser extent MARV entry, in NPC1^−/−^mNPC1 cells was increased in the presence of hTIM1 (right panels of Figs. 8A, B). In contrast, the previously reported non-permissiveness of NPC1^−/−^ cells to EBOV and MARV [15] was not circumvented by hTIM1 overexpression (left panels of Figs. 8A, B). These data indicate that hTIM1 expression cannot overcome NPC1 dependence, confirm the role of NPC1 as a filovirus entry factor and suggest that hTIM1 itself may best be regarded as an attachment factor.

## Discussion

**Figure 9:**
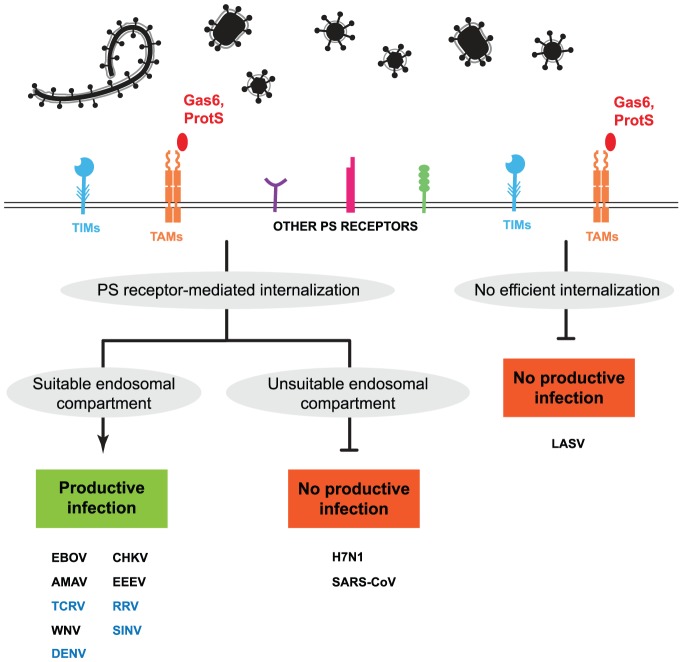
A model of viral PS receptor usage. Human TIM proteins (TIMs) and other PS-binding receptors efficiently, but nonspecifically, promote internalization of various enveloped viruses. The TAM receptors Axl, Mer and Tyro (TAMs) similarly bind and internalize many viruses, albeit indirectly via the PS-binding bridging proteins Gas6 and/or Protein S (Prot S) in serum [35]. For most viruses PS-dependent internalization results in a moderate to strong increase of productive infection. Note that the degree of enhancement will depend on the cellular background in which experiments are performed. However, in the case of other viruses, PS receptor-mediated internalization may lead to a compartment that is not productive for infection. In addition, there is a third class of viruses, which is not efficiently bound and/or internalized by PS receptors. The indicated viruses were categorized based on hTIM1 usage and internalization results obtained with pseudoviruses and VLPs (black) or replication-competent viruses (blue). Note, however, that the consequences or efficiency of internalization can vary depending on the PS receptors and their expression levels.

Our results, summarized in Figure 9, demonstrate that hTIM1 is an efficient attachment factor for a range of enveloped viruses, and imply that hTIM1 promotes infection by associating with PS on the virions. PS dependency of TIM1 is supported by several independent lines of evidence. First, all pseudoviruses capable of using hTIM1 can also be enhanced by at least one other PS receptor, e.g., hTIM4 or hAxl (Fig. 7). Second, a functional, hTIM1 PS-binding domain was an absolute prerequisite for viral hTIM1 usage (Fig. 3). Finally, the interaction between hTIM1 and GP-free virions was sufficient to elicit attachment and internalization, and this internalization was blocked by PS-containing liposomes (Figs. 4 and 5).

Our data are noteworthy and surprising in a few respects. First, the finding that the entry efficiency of a large number of viruses can be enhanced by TIM1 or related receptors suggests that PS receptors may play a more prominent role in viral entry than previously appreciated. This may appear difficult to reconcile with structural studies reporting that the membranes of flavi- and alphaviruses are occluded by tightly-arranged viral glycoproteins. However, flavivirus virions often incorporate immature glycoproteins [57], which could lead to gaps in the glycoprotein coat through which PS becomes accessible. Our findings are consistent with another recent report for a role of TIM proteins in flavivirus entry [58]. Second, as described below, the importance of PS receptors in viral entry is underscored by the fact that several viruses are known to initiate infections in PS receptor-rich cells. Finally, because the exposure of PS on virions is likely a common feature of enveloped viruses [33], it is perhaps most surprising that the entry of a number of viruses remains unaffected by TIM1 expression.

There are several plausible explanations for why not every PS receptor supports the entry of every enveloped virus (Fig. 9). First, PS receptor usage could be affected by the endocytic routes of *bona fide* virus receptors. For example, the six arenaviruses used in Figure 1C can be divided into three groups with respect to TIM1 use: LASV and LCMV, which use alpha-dystroglycan as their cellular receptor [59] only weakly utilize TIM1; MACV and JUNV whose receptor is TfR1 [42], [50] use TIM1 with moderate efficiency; and TCRV and AMAV, which lack a known human receptor, utilize TIM1 with exceptionally high efficiency. For TIM1 to enhance infection, the endocytic pathways of TIM1-mediated internalization probably need to coincide with those of the primary receptors of these viruses. The finding that hTIM1 use by MACV is dependent on the presence of hTfR1 (Fig. 6B) is consistent with this hypothesis. However, the same explanation may not fully describe the clear differences in TIM1 usage among EBOV, MARV, H7N1 and SARS-CoV (Figs. 1 and S1), all of which need access to late endosomes/lysosomes for productive infection. A second possibility, suggested by Morizono and colleagues for hAxl, is that high-affinity receptors may render hTIM1 use ineffective for some viruses [35]. Although appealing, this receptor-affinity hypothesis cannot explain the absence of TIM1 usage by the SARS-CoV SZ isolate with low affinity to hACE2 or the finding that MACV's hTIM1 use was not enhanced upon blockage of the hTfR1-mediated entry route (Fig. 6). A final possibility is that steric hinderance by individual viral entry proteins may affect TIM1-mediated enhancement. Specifically, long or densely packed entry proteins, which are usually covered with glycans, may prevent PS receptors from reaching PS on the viral membrane. Consistent with this hypothesis, truncated hTIM1 variants and hTIM3, which has a much shorter mucin stalk than hTIM1, were used less efficiently than wt hTIM1 by several pseudoviruses (Figs. 7 and S4).

The efficiency with which viruses utilize PS receptors also appears to be dependent on the cellular background. For example, we show that overexpression of hTIM1 had a lesser effect on viral entry in 3T3 compared to 293T cells (Fig. 1). This difference may be due to cell-specific expression of various endogenous PS receptors. Consistent with this explanation, 3T3 cells express Axl [60], [61], while 293T cells do not [11], [35]. In addition, WNV VLPs, which do not efficiently utilize Axl (Fig. 7A), were the only VLP/pseudovirus showing strong TIM1-mediated entry enhancement in 3T3 cells (Fig. 1D). This example emphasizes that results obtained with specific cell lines need to be generalized with caution.

One important question concerning viral PS receptor usage is whether PS receptors are sufficient for productive infection or whether virus-specific receptors are still required. We showed for several pseudoviruses that TIM1-mediated entry depended on the availability of their cellular receptors (Figs. 6B and 8), indicating that TIM1 functions solely as an attachment factor. For instance, TIM1 was unable to enhance the infection of EBOV and MARV pseudoviruses in the absence of the intracellular filovirus entry factor NPC1 (Fig. 8). Also, blocking the accessibility of MACV pseudovirus to its receptor hTfR1 lowered MACV TIM1 usage (Fig. 6B). On the other hand, some viruses were reported to fuse with receptor-free liposomes at low pH [62]–[64], raising the possibility that in a few specific cases PS receptor-mediated internalization may be sufficient for productive infection. Nevertheless, studies using liposomes that are unnaturally enriched with specific lipid components may not accurately represent physiological conditions.

PS receptors form a complementary, widely expressed network of receptors that is characterized by functional rather than structural conservation. These particular features of PS receptors likely contribute to their exploitation by viruses as attachment factors. Notably, several PS receptors, including TIM4, are highly expressed on mammalian macrophages and dendritic cells. These cells play critical roles in the initial stages of infection of filoviruses and flaviviruses in particular [55], [56], [65], and PS receptors may thus play correspondingly important roles in establishing these infections. PS receptors may be more important still for viruses like DENV, WNV, EEEV and CHKV, which are borne by insect vectors. The role of PS as apoptotic marker is conserved in insects [66], and compared to mammalian cells the membranes of insect cells are generally enriched with PE [67], [68], which binds several PS receptors. Thus mosquito-delivered virions may especially benefit from PS receptor-mediated enhancement of infection.

In conclusion, our results indicate that hTIM1, hTIM4, hAxl and potentially other PS-binding receptors can enhance the entry of a number of highly divergent viruses. As demonstrated for hTIM1, the enhancement conferred by all of these receptors is likely PS dependent and does not require any viral entry protein. Accordingly, these proteins cannot properly be described as viral receptors, although the nature of the viral entry protein clearly impacts the relevance of PS receptors to infection. In some cases, PS receptors may play critical roles in establishing or maintaining an *in vivo* infection, which could affect disease severity. Thus our results support the proposal of Soares et al., demonstrated for Pichinde virus and mouse cytomegalovirus [33], that therapeutic strategies targeting PS and other anionic phospholipids may be broadly effective against a wide range of viruses.

## Supporting Information

Figure S1 **Supplemental studies of exogenous hTIM1 use in 293T and 3T3 cells.** (A & C) hTIM1 use in 293T or 3T3 cells represented using % positive cells. In parallel with the mean fluorescence intensity (m.f.i.), infection levels were assessed using the percentage of GFP positive cells. Note that these graphs also show data for MLV pseudoviruses bearing no viral entry glycoproteins (no GP). Figure shows representative results of one of three experiments performed in duplicates. (B & D) In parallel with the short infection times used for assessing hTIM1 usage in Figures 1C and D, 293T and 3T3 cells were subject to a longer infection (4 h for 293T and 12 h for 3T3) to assess whether pseudovirus titers might be limiting, especially for viruses showing low or no TIM1 use. Figure shows representative results from one of three experiments performed in duplicates. (E & F) SARS (Tor2) pseudovirus entry is not enhanced by exogenous hTIM1 in 293T or 3T3 cells. SARS (Tor2) pseudovirus entry into 293T cells expressing the empty vector (mock), hTIM1 or the SARS-CoV receptor hACE2 was assessed as in Figure 1C. Since 293T or 3T3 cells are completely refractory to SARS pseudovirus entry in the absence of exogenous hACE2, both positive *and* negative control cells were used, making it necessary to separate this data from Figs. 1C and D. Figure shows mean+SD of three independent, duplicated experiments.
(EPS)

Figure S2 **Supplemental studies of hTIM1 use by infectious viruses.** 293T cells transduced with hTIM1 or hACE2 were incubated for 6 h with infectious TCRV (A), RRV (B) or SINV (C), or for 1 h with infectious DENV (D), washed, and further incubated for one, two or three consecutive days. At the indicated days, cells were trypsinized, stained either with immune ascitic fluids (A–C) or with anti-DENV antibody (D), and infection levels were measured by flow cytometry. Figures show a representative of two duplicated experiments (A) or mean ± SD of two duplicated experiments (B–D).
(EPS)

Figure S3 **PE-containing liposomes also block hTIM1-mediated enhancement of viral entry.** 293T cells transduced with hACE2 (mock) or hTIM1 were preincubated for 20 min at room temperature with medium alone (none) or with liposomes consisting of either 50% PE and 50% PC (PE/PC) or PC alone (PC). Pseudoviruses or WNV VLPs were then added for a 30 min infection at 37°C, after which unbound liposomes and viruses were washed off and cells supplemented with fresh medium. Infection levels were assessed the following day by measuring GFP expression, and normalized to those of untreated hTIM1-expressing cells. Figure shows mean+SD from three independent, duplicated experiments.
(EPS)

Figure S4 **Contribution of stalk length and O-glycans of hTIMs to virus entry.** (A) Schematic representation of the three human TIM proteins and stalk-truncated hTIM1 variants used in B–C. hTIM3 differs from the other TIMs because its stalk is shorter and because it has a dramatically reduced number of O-glycans [18]. The two truncation variants of hTIM1, Δ131-221 and Δ197-287 hTIM1, were made to resemble hTIM3 in stalk length and number of O-glycosylation sites. (B) Expression levels of stalk-truncated and wt hTIM1 are comparable. 293T cells were transfected with plasmids expressing hACE2 (mock), Δ131-221 hTIM1, Δ197-287 hTIM1 or wt hTIM1. 48 h later cells were stained with the anti-hTIM1 antibody 3D1, which binds the IgV head domain of hTIM1 [21]. Figure shows representative results of one of three experiments performed in duplicates. (C) Usage of stalk-truncated hTIM1 variants in 293T cells. The same cells as in B were infected with the indicated pseudoviruses carrying a GFP reporter gene. Infection levels were assessed the following day by flow cytometry and normalized to those of wt hTIM1-expressing cells. Figure shows representative results of one of three experiments performed in duplicates.
(EPS)

Text S1 **Molecular causes underlying the inefficient viral usage of TIM3.**
(DOC)
