# Supplementary material for: TIM-family Proteins Promote Infection of Multiple Enveloped Viruses through Virion-associated Phosphatidylserine
Source: PLoS Pathog. 2013 Mar 28;9(3):e1003232. doi: 10.1371/journal.ppat.1003232 (PMC3610696; doi:10.1371/journal.ppat.1003232)
Supplement: Text S1 — Molecular causes underlying the inefficient viral usage of TIM3. (DOC) [file ppat.1003232.s005.doc]

**SUPPLEMENTARY TEXT S1**

**Molecular causes underlying the inefficient viral usage of TIM3**

Although hTIM3 binds PS, it differs from hTIM1 and 4 in several aspects that could explain why it does not efficiently support viral entry. First, based on results obtained with the corresponding mouse TIM orthologs, its affinity for PS is lower than that of TIM1 and 4 [1]. Second, as illustrated in Figure S4, its stalk is much shorter than that of the other TIMs, which may prevent its PS-binding IgV domain from effectively reaching the GP-studded viral membranes. Finally, the stalk of TIM3 bears only a few O-glycosylation sites, whereas those of TIM1 and 4 are heavily covered with O-linked carbohydrates that could potentially participate in virus binding. To distinguish among these possible explanations, we produced two stalk-truncated hTIM1 variants, each similar in stalk length, but with very different numbers of O-glycosylation sites (Fig. S4A). These hTIM1 variants were expressed in 293T cells along with wt hTIM1 and a control receptor and infected with various hTIM1-using pseudoviruses, as well as with H7N1 pseudoviruses as a negative control. As shown in Figures S4B and S4C, truncation of the hTIM1 stalk affected entry of hTIM1-using pseudoviruses to varying degrees, with a considerable effect on EBOV, CHKV and EEEV and marginal effects on AMAV and TCRV. Hence, while a long stalk seems to help support the entry of some pseudoviruses, it is not a major determinant of TIM protein usage for others. Our findings also show that the O-glycans on the stalk play little or no role in determining viral TIM-protein usage, since the entry enhancements conferred by the heavily glycosylated ∆197-287 and the sparsely glycosylated ∆131-221 hTIM1 variants were similar when taking the minor difference in expression into account.

**SUPPLEMENTARY REFERENCES**

1. DeKruyff RH, Bu X, Ballesteros A, Santiago C, Chim YL, et al. (2010) T cell/transmembrane, Ig, and mucin-3 allelic variants differentially recognize phosphatidylserine and mediate phagocytosis of apoptotic cells. J Immunol 184: 1918-1930.
